# Chemical Experiments and Observations, on the Extraction of Sugar and Syrup from Indigenous Plants

**Published:** 1800-10

**Authors:** Sigism. Henry Hermbstaedt


### *Chemical Experiments and Observations, on the Extraction of Sugar and Syrup from Indigenous Plants. By* SIGISM. HENRY HERMBSTAEDT.

[ Concluded from our laft, pp. 246—252. ]

11. *Experiments with Carrots, (Daucus Carotta, L.)*

BY cutting carrots into pieces, and boiling them with water, a fweet liquor is got, of which, by evaporation, country people prepare a juice, which they call carrot-fyrup, and eat either with bread, or ufe it inftead of fugar, and by which common fyrup is fometimes adulterated. But as the juice obtained in this way is ftill too mucilaginous to be ufed as a fyrup, I operated in the following manner: A bufhel of carrots being freed from their rind, were ground upon a grater, and the juice expreffed. It was then boiled, clarified with the white of eggs, and afterwards thickened to the confiftency of a fyrup; and of this, fix pounds and a half were obtained, which though of a pretty agreeable tafte, was by far inferior to that of both beets. It was impoffible to draw any faccharine cryftals from it, and the alkohol did but extract a fubftance very much like manna.

12. *Experiments with Common White Turnips, (Brafica Rapa.)*

Twelve turnips were ground upon a grater, being freed from the outer rind, and gave by expreffion a fweetifh juice. This being clarified, and ftrained through a woollen cloth, was thickened to a fyrup of an agreeable tafte, and though inferior to that of the beets, it is equal to common fyrup. By another experiment, I found that from one hundred and fixteen pounds of turnips, eight pounds of fyrup may be obtained, in which, after fome months, cryftals of brown fugar had formed; but they feparate with difficulty from the remaining parts.

13. *Experiments with the Cole Rape, (Braffica Napobraffica.)*

The fucculency of this turnip, and the fweetnefs of its juice, induced me to undertake the fame experiments with it; fixty roots were therefore ground and expreffed, and gave twenty-two quarts of fweetifh juice, which, however, tafted a little acrid. By feething it, a great deal of its impurities feparated, and the fluid became quite clear. Being filtered, it was mixed with twenty quarts of lime-water and gently boiled, by which the acrid tafte went off, and a fyrup was got, that had, in every refpect, a great likenefs with that of the black birch, and may be ufed inftead of common fyrup. To obtain fugar from it, a portion was evaporated in a glafs veffel; and after fome weeks, fmall cryftals of it were formed, whofe quantity I could not determine.

14. *Experiments with the Skirret, (Sium Sifarum, L.)*

Mr. Marggraff obtained from a pound of frefh fkirret roots, four ounces and a half of dried; and half a pound of dried roots gave three drachms of a pure fugar. My experiments were only made for the purpofe of obtaining a fyrup from it, as the extraction of fugar is fubject to many difficulties, on account count of their containing too great a quantity of farinaceous matter. A bushel of these roots, which weighed about twenty-six pounds, was pounded in a mortar, and afterwards repeatedly expressed, with the addition of some cold water, by which means a turbid sweet juice was obtained By exposing it in a cool place a quantity of farinaceous matter separated, and the liquor became clear. Being clarified with the white of eggs, and thickened, five pounds of a very agreeable syrup of a light brown colour were got; but it would prove too expensive for common use.

15. *Experiments with Parsnips, (Pastinaca Sativa, L.)*

The sweetness of these roots induced me to undertake a similar operation with them, though Marggraff had remarked, that but little sugar is to be obtained from them. A bushel of them, weighing twenty-four pounds, was accordingly treated in the same way as the former, and five pounds and a half of an agreeable syrup were extracted, which however had not quite lost the specific taste of parsnips.

These are the only experiments my time has hitherto allowed me to undertake; I have not yet been able to determine the relative proportion of sugar that may be obtained from each of those vegetables, but I shall endeavour to make some experiments by which this may be ascertained.

